# Chronic ethanol exposure enhances the aggressiveness of breast cancer: the role of p38γ

**DOI:** 10.18632/oncotarget.6508

**Published:** 2015-12-07

**Authors:** Mei Xu, Siying Wang, Zhenhua Ren, Jacqueline A. Frank, Xiuwei H. Yang, Zhuo Zhang, Zun-ji Ke, Xianglin Shi, Jia Luo

**Affiliations:** ^1^ Department of Pharmacology and Nutritional Sciences, University of Kentucky College of Medicine, Lexington, KY 40536, USA; ^2^ Pathophysiological Department, School of Basic Medicine, Anhui Medical University, Hefei, Anhui 23002, China; ^3^ Department of Toxicology and Cancer Biology, University of Kentucky College of Medicine, Lexington, KY 40536, USA; ^4^ Department of Biochemistry, Shanghai University of Traditional Chinese Medicine, Shanghai 201203, China

**Keywords:** alcohol abuse, cancer stem cells, metastasis, mammary tumor

## Abstract

Both epidemiological and experimental studies suggest that ethanol may enhance aggressiveness of breast cancer. We have previously demonstrated that short term exposure to ethanol (12–48 hours) increased migration/invasion in breast cancer cells overexpressing ErbB2, but not in breast cancer cells with low expression of ErbB2, such as MCF7, BT20 and T47D breast cancer cells. In this study, we showed that chronic ethanol exposure transformed breast cancer cells that were not responsive to short term ethanol treatment to a more aggressive phenotype. Chronic ethanol exposure (10 days - 2 months) at 100 (22 mM) or 200 mg/dl (44 mM) caused the scattering of MCF7, BT20 and T47D cell colonies in a 3-dimension culture system. Chronic ethanol exposure also increased colony formation in an anchorage-independent condition and stimulated cell invasion/migration. Chronic ethanol exposure increased cancer stem-like cell (CSC) population by more than 20 folds. Breast cancer cells exposed to ethanol *in vitro* displayed a much higher growth rate and metastasis in mice. Ethanol selectively activated p38γ MAPK and RhoC but not p38α/β in a concentration-dependent manner. SP-MCF7 cells, a derivative of MCF7 cells which compose mainly CSC expressed high levels of phosphorylated p38γ MAPK. Knocking-down p38γ MAPK blocked ethanol-induced RhoC activation, cell scattering, invasion/migration and ethanol-increased CSC population. Furthermore, knocking-down p38γ MAPK mitigated ethanol-induced tumor growth and metastasis in mice. These results suggest that chronic ethanol exposure can enhance the aggressiveness of breast cancer by activating p38γ MAPK/RhoC pathway.

## INTRODUCTION

Breast cancer is the second leading cause of cancer among American women [1]. Although the exact etiology for breast cancer initiation and development is unclear, environmental factors play an important role. Epidemiological studies indicate that alcohol consumption significantly increases the risk for breast cancer in a concentration- and duration-dependent manner [2–4]. In addition to the promotion of breast cancer carcinogenesis, alcohol may also enhance the growth of existing breast tumors and increase the aggressiveness of breast cancer cells to invade and metastasize [5–7]. Nonetheless, the underlying mechanisms are unknown. Various experimental models have been employed to investigate the effect of alcohol on breast cancer. The epidemiological data are supported by experimental studies which show that alcohol promotes mammary tumorigenesis/metastasis in animals, stimulates migration/invasion of breast tumor cells and enhances the expression of markers for epithelial-mesenchymal transition in cell culture systems [8–22].

Previous studies show that short-term treatment of alcohol, that is, exposure for 12–48 hours, is sufficient to alter the behaviors of breast cancer cells, resulting in enhanced aggressiveness. However, breast cancer cells display differential sensitivity to alcohol exposure, that is, some cells are much more sensitive to alcohol exposure and some are not responsive [15]. For example, the short-term alcohol exposure stimulates the migration/invasion of breast cancer cells expressing high levels of ErbB2/HER2 [8, 12–15]. This may result from alcohol-induced amplification of ERbB2 signaling. However, some breast cancer cells, particularly the breast cells that express low levels of ErbB2, such as MCF7, T47D and BT20 cells, are quite resistant to alcohol exposure; the short-term treatment of alcohol has little effect on their migration/invasion behavior. We hypothesize that a long-term treatment of alcohol is required to convert these “alcohol-resistant” cells to a more aggressive phenotype. In this study, we showed that long- term treatment of alcohol, 10 days-2 months, significantly increased the migration/invasion, cancer stem-like cell population and tumorigenicity in “alcohol-resistant” cells *in vitro* and *in vivo*. We further demonstrated that p38γ MAPK played an important role in alcohol-promoted aggressiveness in these cells. p38γ MAPK is one of four members of the p38 MAPK family [23]. Recent studies indicate that p38γ MAPK is implicated in breast cancer progression and aggressiveness [24]. We showed here that alcohol selectively activated p38γ MAPK and its down-stream effector, RhoC, resulting in enhanced aggressiveness of breast cancer cells.

## RESULTS

### Ethanol converts less aggressive breast cancer cells to a more aggressive phenotype

**Figure 1:**
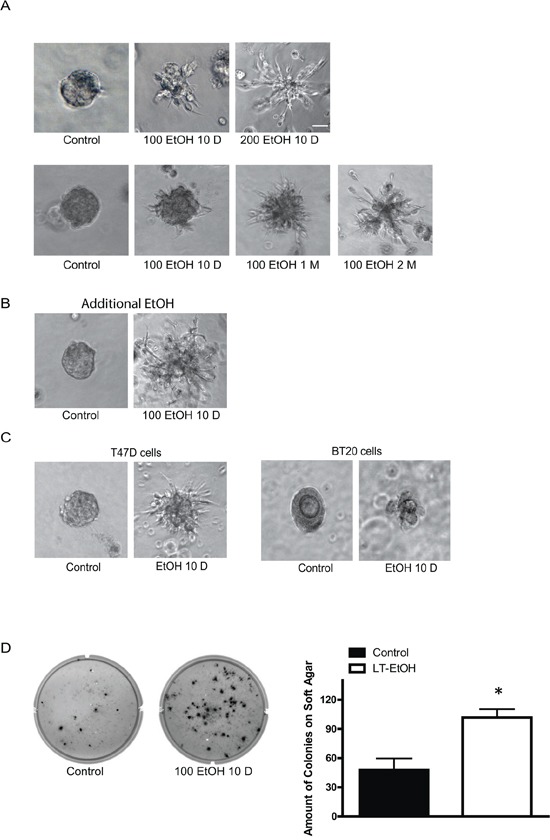
Effects of chronic ethanol exposure on the malignancy of breast cancer cells **A.** MCF7 cells were exposed to ethanol (0, 100, or 200 mg/dl, i.e., 0, 22 or 44 mM) for 10 days, 1 month or 2 months. After ethanol exposure, single cell suspension (10^3^ cells/well) was cultured in Matrigel and maintained in an ethanol free environment for 10 days as described in Materials and Methods **B.** After chronic exposure to ethanol, single cell suspension was cultured in Matrigel and maintained with ethanol (100 mg/dl) for 10 days. **C.** T47D or BT20 cells were exposed to ethanol (0 or 100 mg/dl) for 10 days, then cultured in ethanol-free Matrigel for an additional 10 days. **D.** MCF7 cells were exposed to ethanol (0 or 100 mg/dl) for 10 days and then maintained in an ethanol-free soft agar assay for a month. Number of colonies/well was counted. Each data point was the mean ± SEM of three independent experiments. * denotes significant difference from controls, bar = 50 μm.

**Figure 2:**
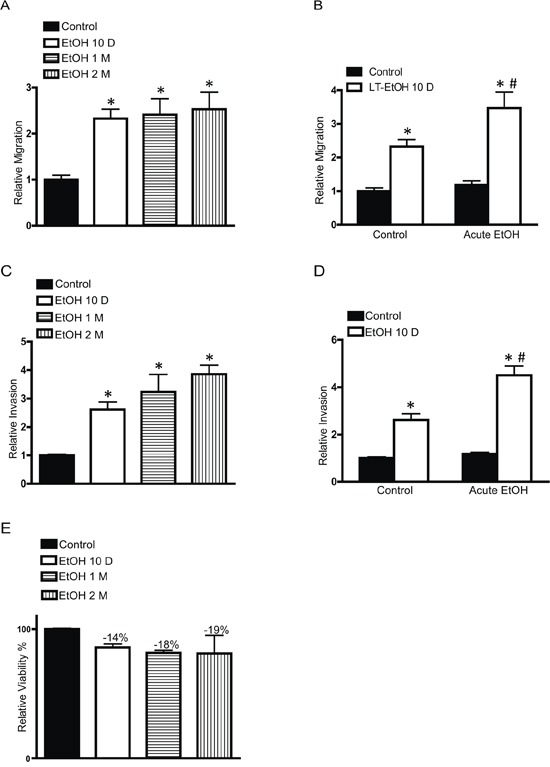
Effects of chronic ethanol exposure on cell migration/invasion MCF7 cells were exposed to ethanol (0 or 100 mg/dl) for 10 days, 1 month or 2 months. After that, the migration **A.** and invasion **C.** were assayed during a 12 hour period in an ethanol free environment. In other experiments, MCF7 cells were exposed to ethanol (0 or 100 mg/dl) for 10 days, the migration **B.** and invasion **D.** were assayed in the presence or absence of ethanol (100 mg/dl) during a 12 hour period. After ethanol exposure, cell viability was determined by MTT assay as described in the Experimental procedures **E.** Each data point was the mean ± SEM of three independent experiments and presented relative to the control values. * denotes significant difference from no ethanol-treated control groups. # denotes significant difference from 10 day-ethanol-treated groups.

**Figure 3:**
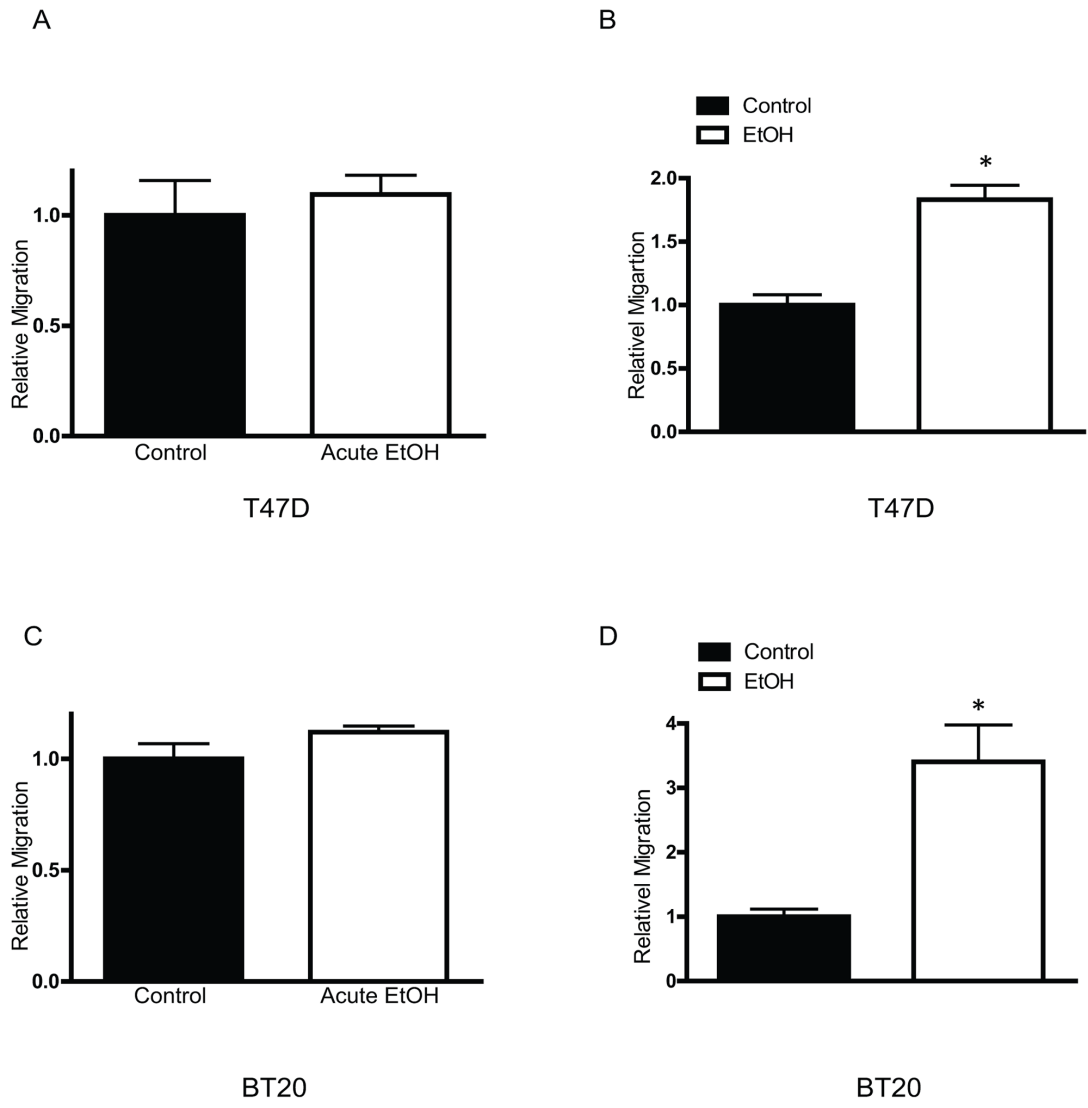
Effects of chronic ethanol exposure on T47D and BT20 cells Equal amounts of T47D **A.** or BT20 **C.** cells were assayed for their migration ability with short term ethanol (0 or 100 mg/dl) exposure (12 hours). T47D **B.** or BT20 cells **D.** were exposed to ethanol (0 or 100 mg/dl) for 10 days, then equal amount of cells were assayed for their migration ability during a 12 hour period in an ethanol free environment. Each data point was the mean ± SEM of three independent experiments and presented relative to the control values. * denotes significant difference from no ethanol-treated control groups.

MCF7 cells are a less aggressive breast cancer line and display modest invasion and metastatic potential. Our previous study indicated that the short term ethanol exposure (12–48 hours) had little effect on the behaviors of MCF7 breast cancer cells [15]. We first examined the effect of chronic ethanol exposure on MCF7 cells. MCF7 cells were exposed to ethanol (100 or 200 mg/dl) for 10 days∼2 months at concentrations relevant to human alcohol consumption and then grown in an ethanol-free 3-D Matrigel system for an additional 10 days. It was reported that aggressive breast cancer cells, such as MB231, have a property to grow to scattering spheroids in 3-D culture system [24]. Ethanol increased the scattering of MCF7 cells in a concentration- and duration-dependent manner (Figure 1A) which indicated an enhanced aggressiveness. The continuous presence of ethanol after chronic exposure further increased the scattering (Figure 1B). A similar effect of ethanol on T47D and BT20 breast cancer cells was observed (Figure 1C). Both T47D and BT20 cells are less aggressive and barely respond to short term ethanol exposure [14, 15], but chronic ethanol exposure increased the scattering of these cells (Figure 1C). Noscattering spheroids were observed in control MCF7, T47D and BT20 cells. Chronic ethanol exposure-enhanced malignancy was also supported by the increase in anchorage-independent colony formation (Figure 1D). In addition, chronic ethanol exposure increased the migration/invasion of MCF7 cells (Figure 2). In this experiment, MCF7 cells were exposed to ethanol (100 mg/dl) for 10 days, 1 month or 2 months, and then assayed for their migration/invasion during a 12 hour-period in an ethanol free environment. Chronic ethanol exposure significantly increased migratory (Figure 2A) and invasive potential of MCF7 cells (Figure 2C); however, only the effect of ethanol on invasion was duration-dependent. Continuous ethanol presence in the medium further increased the migration/invasion (Figure 2B and 2D). Once again, it was confirmed that short-term ethanol exposure failed to alter migration/invasion of MCF7 cells (comparing the solid bars in Figure 2B and 2D: control versus acute ethanol exposure). Chronic ethanol exposure caused a modest decrease in cell number; this may result from either a slight inhibition of cell proliferation or decrease in cell viability (Figure 2E). Next, we tested the effect of ethanol on T47D and BT20 cells. Similar to MCF7 cells, we have previously demonstrated that both T47D and BT20 cells were insensitive to acute ethanol exposure due to low ErbB2 expression [15]. Here, we confirmed that short-term ethanol exposure (12 hours) did not affect the migration of T47D and BT20 cells (Figure 3A and 3C); however, chronic ethanol exposure (10 days) significantly increased the migration of these cells (Figure 3B and 3D).

**Figure 4:**
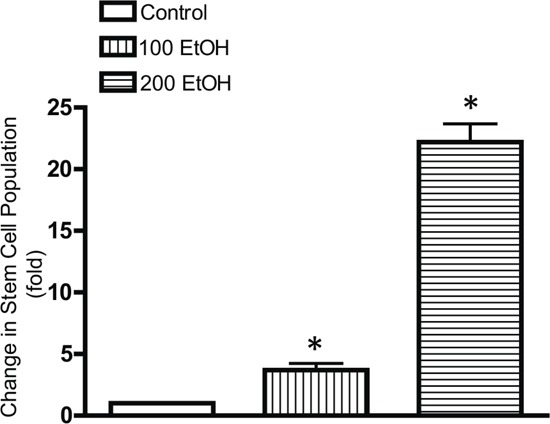
Effects of chronic ethanol exposure on stem-like cell population MCF7 cells were exposed to ethanol (0, 100 or 200 mg/dl) for 10 days, and then stem-like cell population was determined by the ALDEFLUOR as described in Materials and Methods. Data were presented as fold increase relative to the controls. * denotes significant difference from control groups.

We further demonstrated that chronic ethanol exposure significantly increased cancer stem-like cell population (Figure 4). In this experiment, MCF7 cells were exposed to ethanol (100 or 200 mg/dl) for 10 days and then evaluated for the stem-like cell population. Ethanol caused a concentration-dependent increase in stem-like cell population.

### Breast cancer cells pre-exposed to ethanol display higher tumorigenicity *in vivo*

**Figure 5:**
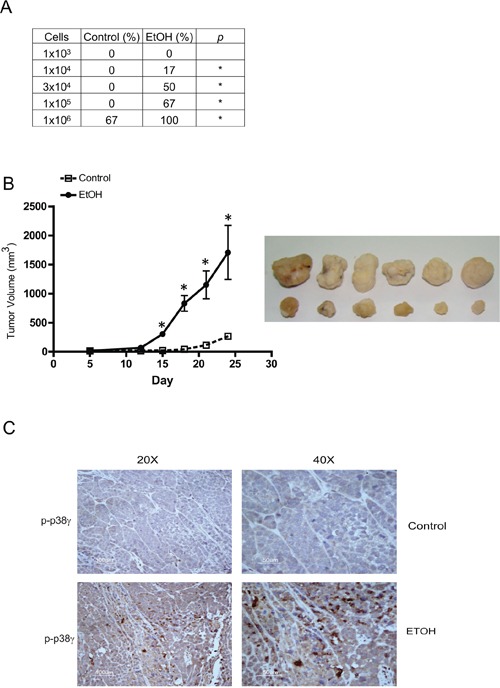
Effects of chronic ethanol exposure on tumorigenesis *in vivo* MCF7 cells were exposed to ethanol (0 or 100 mg/dl) for a month, then xenografted into nude mice as described in the Experimental procedures. **A.** Ethanol-pretreated cells ranging from 10^3^ to 10^6^ cells/100 μl in PBS were subcutaneously inoculated into the lower flank of nude mice (*n* = 6). One month after inoculation, tumorigenicity was evaluated and presented as percentage of the original inoculation. *p < 0.05. **B.** Tumor sizewas measured weekly and tumor volume (mm^3^) was calculated as described in the Experimental procedures. *p < 0.05. **C.** Tumor tissues from control or ethanol-exposed groups were fixed, sectioned and processed for IHC staining of phospho-p38γ (p-p38γ).

To confirm that chronic ethanol exposure indeed converted breast cancer cells to a more malignant phenotype, we treated MCF7 cells with ethanol (0 or 100 mg/dl) for a month and then xenografted these cells in to nude mice. Our data showed that MCF7 cells that were pretreated with ethanol exhibited significantly higher tumorigenicity (Figure 5A) and tumor growth rate (Figure 5B).

### Chronic ethanol exposure selectively activates p38γ MAPK

**Figure 6:**
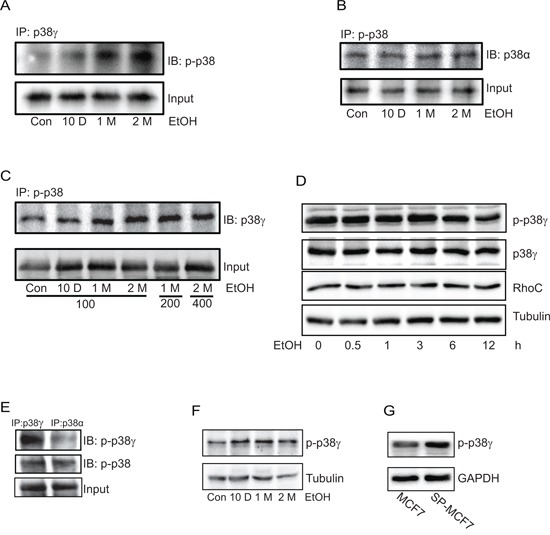
Effect of chronic ethanol exposure on the phosphorylation of p38γ MAPK MCF7 cells were exposed to ethanol (0 or 100 mg/dl) for 10 days, 1 month or 2 months. **A.** Cell lysates were collected and then equal amount of proteins were immuoprecipitated (IP) with an anti-p38γ MAPK antibody and then immunoblotted (IB) with an antibody directed against pan phosphorylated p38 MAPK (p-p38). **B.** Proteins were IP with an anti p-p38 antibody and then IB with an anti-p38α antibody. **C.** MCF7 cells were exposed to ethanol (0, 100, 200 or 400 mg/dl) for indicated times, then proteins were collected and IP with an anti-p-p38 antibody and IB with an anti-p38γ MAPK antibody. **D.** MCF7 cells were exposed to ethanol (100 mg/dl) for 0.5–12 hours. The expression of phosphorylated p38γ MAPK, total p38γ MAPK and RhoC was determined by immunoblotting. **E.** Equal amount of proteins were IP with p38γ or p38α, and then IB with either a commercial anti-pan phosphorylated p38 antibody (p-p38) or a specific anti-phosphorylated-p38γ antibody (p-p38γ) (21st Century Biochemical, please see Materials and Methods). **F.** The same protein samples described on panel A was analyzed with immunoblotting using the specific anti-p-p38γ MAPK antibody. **G.** The expression of phosphorylated p38γ in MCF7 and SP-MCF7 cells was evaluated using a specific anti-p-p38γ antibody as described above. All experiments were replicated at least three times.

p38γ MAPK has been implicated in the aggressiveness of breast cancer cells (32). In animal studies, we showed that tumors developed by ethanol pre-exposed MCF7 cells exhibited higher expression of phospho-p38γ MAPK (Figure 5C), suggesting that p38γ MAPK may be involved in ethanol-promoted aggressiveness. We first investigated whether chronic ethanol exposure activated p38γ MAPK. Using the immunoprecipitation assay withcommercial antibodies, we showed that chronic ethanol exposure increased the phosphorylation of p38γ MAPK in MCF7 cells, but not other isoforms of p38 MAPK (Figures 6A–6C). Only results on p38α are presented and the data on other isoforms are not shown. In contrast, the short term ethanol exposure (0.5–12 hours) did not alter the phosphorylation of p38γ MAPK (Figure 6D). To further validate the finding, we generated a phospho-specific antibody directed against p38γ MAPK with the assistance of 21st Century Biochemical (Marlboro, MA). This antibody was specific for phospho-p38γ MAPK and did not cross react with other p38 MAPK isoforms (Figure 6E). Using this antibody, we confirmed that chronic ethanol exposure specifically increased the phosphorylation of p38γ MAPK (Figure 6F). Using this antibody, we compared the levels of phosphorylated p38γ MAPK between MCF7 cells and its derivative, SP-MCF7 cells, a Hoechst dye excluding mammary cell subline which have more cancer stem-like cell population [25–27]. As shown in Figure 6G, SP-MCF7 cells expressed more phosphorylated p38γ MAPK than MCF7 cells.

### p38γ MAPK mediates ethanol-increased aggressiveness of breast cancer cells

**Figure 7:**
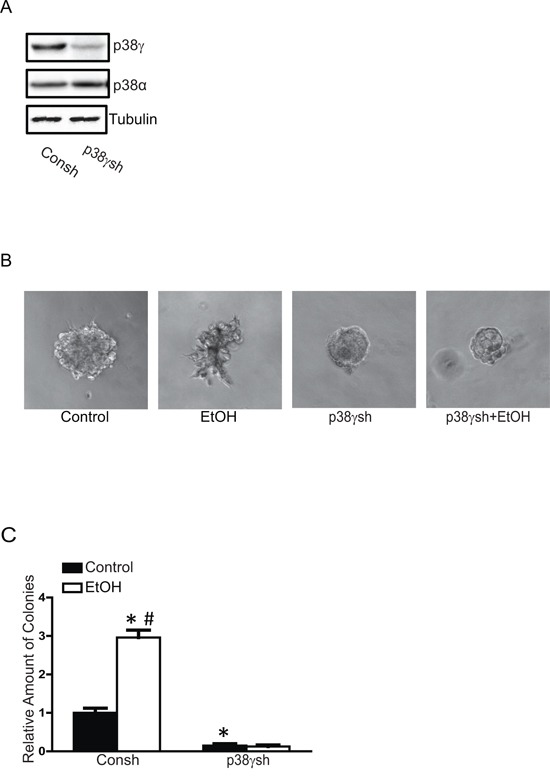
Effects of p38γ knock-down on ethanol-induced malignancy We have established MCF7 cells stably expressing control shRNA (Consh) and p38γ shRNA (p38γsh) as described in the Materials and Methods. The expression of p38γ and p38α was examined with immunoblotting **A.** These cells were exposed to ethanol (100 mg/dl) for 10 days then assayed for cell scattering in a 3-D culture system **B.** and anchorage-independent colonies formation **C.** The results were presented relative to the controls. Each data point was the mean ± SEM of three experiments. * denotes significant difference from control groups. # denotes significant difference from p38γ shRNA EtOH groups.

**Figure 8:**
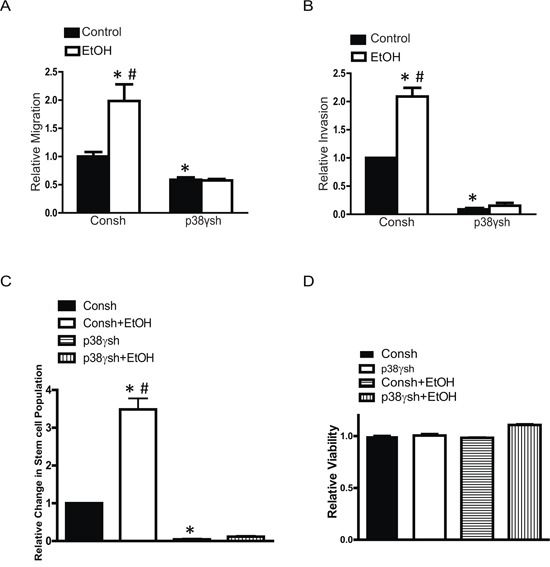
Effects of p38γ knock-down on ethanol-induced migration/invasion and cancer stem-like cell population MCF7 cells stably expressing control shRNA (Consh) and p38γ shRNA (p38γsh) were exposed to ethanol (0 or 100 mg/dl) for 10 days. After that, cell migration **A.** invasion **B.** cancer stem-like cell population **C.** and cell viability **D.** were evaluated as described in the Materials and Methods. The results were expressed relative to the controls. Each data point was the mean ± SEM of three experiments. * denotes significant difference from control groups. # denotes significant difference from ethanol-treated p38γsh groups.

To confirm the involvement of p38γ MAPK in ethanol-enhanced aggressiveness of breast cancer cells, we established MCF7 cells with a stable expression of either control shRNA (Consh) or shRNA for p38γ MAPK (p38γsh) (Figure 7A). Knocking down p38γ MAPK inhibited ethanol-induced cell scattering in the 3-D culture system (Figure 7B) and the anchorage-independent colony formation (Figure 7C). Knocking down p38γ MAPK also blocked ethanol-stimulated cell migration (Figure 8A) and invasion (Figure 8B). Furthermore, knocking down p38γ MAPK inhibited an ethanol-induced increase in stem-like cell population (Figure 8C). Down-regulation of p38γ MAPK did not alter cell viability (Figure 8D).

**Figure 9:**
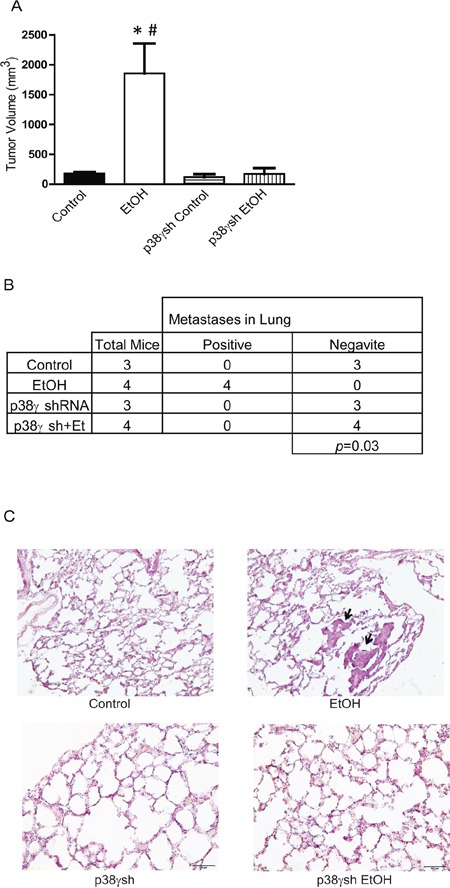
Effects of p38γ knock-down on ethanol-induced tumor growth and metastasis MCF7 cells stably expressing control shRNA and shRNA for p38γ MAPK (p38γsh) were exposed to ethanol (0 or 100 mg/dl) for a month, then 5 × 10^6^ cells (in 100 ul PBS) were inoculated into nude mice on both sides of the lower flank as described in the Materials and Methods. **A.** Four weeks after inoculation, the tumors were measured and the average volume was calculated. * denotes significant difference from mice inoculated with MCF7 cells. # denotes significant difference from mice inoculated with MCF7 cells expressing p38γ shRNA. **B.** At completion of experiments, mice were sacrificed and analyzed for tumor metastasis as described in the Materials and Methods. **C.** Lung tissues were fixed, sectioned and stained. The image shows metastatic carcinomas in the lungs of mice that were inoculated with ethanol-treated MCF7 cells.

The critical role of ethanol-induced aggressiveness of breast cancer was further confirmed in animal studies. MCF7 cells expressing control shRNA or shRNA for p38γ were exposed to ethanol (100 mg/dl) for a month and then inoculated to nude mice. As shown in Figure 9A, expression of shRNA for p38γ MAPK significantly blocked ethanol-stimulated tumor growth in nude mice. Furthermore, the knock down p38γ MAPK by the shRNA blocked ethanol-induced lung metastasis (Figures 9B and 9C).

### Mechanisms underlying p38γ MAPK-mediated aggressiveness of breast cancer cells

**Figure 10:**
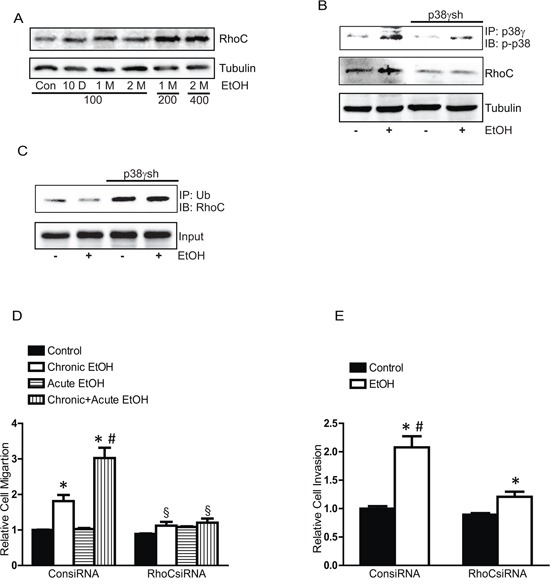
Effects of chronic ethanol exposure on RhoC expression **A.** MCF7 cells were exposed to ethanol (0, 100, 200 or 400 mg/dl) for 10 days, 1 month or 2 months, then the expression of RhoC was examined by immunoblotting. **B.** MCF7 cells stably expressing control shRNA and p38γ shRNA (p38γsh) were exposed to ethanol (0 or 100 mg/dl) for 10 days. After that, cell lysates were IP with an anti-p38γ antibody, and then IB with an anti p-p38 MAPK antibody. The expression of RhoC was examined. **C.** Cell lysates were IP with an anti-ubiquitin antibody and IB with an anti-RhoC antibody. **D.** MCF7 cells were exposed to ethanol (0 or 100 mg/dl) for 10 days, and then treated with either control siRNA or RhoC siRNA for 48 hours. After that, cells were assayed for the migration during a 12-hour-period with/without ethanol (100 mg/dl). **E.** Cell invasion was evaluated. Each data point was the mean ± SEM of three experiments and presented relative to the controls values. * denotes significant difference from the controls. # denotes significant difference from long term EtOH (LT EtOH). δ denotes significant difference from corresponding controls.

RhoC is a key protein that mediates the cell shape and motility and is implicated in the aggressiveness of breast cancer [28, 29]. It has been reported that p38γ MAPK may regulate the levels of RhoC by controlling its degradation [24]. We first sought to determine whether ethanol altered the levels of RhoC and then to investigate the role of p38γ MAPK in ethanol-induced alteration of RhoC. As shown in Figure 10A, chronic ethanol exposure increased the levels of RhoC in MCF7 cells. Knocking down p38γ MAPK blocked ethanol-mediated increase of RhoC (Figure 10B). p38γ MAPK may alter RhoC levels by regulating its ubiquitin-dependent degradation [24]. We showed that ethanol decreased the ubiquitination of RhoC and down-regulation of p38γ MAPK increased the ubiquitination of RhoC (Figure 10C). Together, these results suggested that ethanol-mediated p38γ MAPK activation inhibited ubiquitination of RhoC, resulting in increased in RhoC levels. To confirm the role of RhoC in ethanol-induced aggressiveness, we knocked down RhoC expression by specific siRNA and examined the effect of ethanol on the migration/invasion of MCF7 cells. As shown in Figures 10D and 10E, RhoC siRNA effectively inhibited ethanol-induced migration and invasion, confirming that it played a critical role in ethanol-induced aggressiveness of breast cancer cells.

## DISCUSSION

We demonstrate here that chronic exposure to ethanol can transform breast cancer cells that do not normally respond to short-term exposure to a more aggressive phenotype. This is demonstrated by a significant increase in the scattering of these cells in the aggressive 3-dimensional growth, more colony formation in an anchorage-independent condition, enhanced cell invasion/migration and higher CSC population following chronic exposure to ethanol (10 days – 2 months). This is also evident by the finding that the breast cancer cells chronically exposed to ethanol *in vitro* display a much higher rate of growth and metastasis in mice. It appears that p38γ MAPK plays an important role in ethanol-promoted aggressiveness. Knocking-down p38γ MAPK blocks ethanol-stimulated cell scattering, invasion/migration, an increase in CSC population as well as *in vivo* tumor growth and metastasis.

We have previously demonstrated that short term exposure to ethanol (12–48 hours) increased migration/invasion in breast cancer cells overexpressing ErbB2, but not in breast cancer cells with low expression of ErbB2, such as MCF7, BT20 and T47D breast cancer cells [15]. The current study indicates that given enough time ethanol can stimulate breast cancer cells that are not responsive to short term ethanol exposure. It appears the effect of ethanol is not reversible at least in the present experimental models. This is demonstrated by the results that the effect of ethanol on cell scattering, colony formation, migration/invasion and CSC population is persistent even after the removal of ethanol. Similarly, it appears that breast cancer cells treated with ethanol *in vitro* have acquired greater potential to grow and metastasize in mice that are free of ethanol exposure. It is noted that continuous presence of ethanol further enhances the stimulatory effect of chronic ethanol exposure (Figures 1B, 2B and 2D).

We have previously shown that short-term exposure to ethanol can stimulate the phosphorylation of p38 MAPK in breast cancer cells overexpressing ErbB2, but not in cells expressing low levels of ErbB2 [15]. However, in that study we did not differentiate which isoforms of p38 MAPK were being activated by ethanol. Consistent with the previous finding, our current results confirm that short-term ethanol exposure does not elicit phosphorylation of p38 MAPK in MCF7 cells (Figure 6D). Instead, we show that chronic ethanol exposure specifically increases the phosphorylation of p38γ MAPK but not other isoforms of p38 (Figure 6). The effect of ethanol on p38γ MAPK phosphorylation parallels its effect on cell behaviors, that is, only chronic exposure to ethanol has stimulatory effects on cell scattering, colony formation, migration/invasion and CSC population.

The p38 MAPK family is comprised of four members, p38*α*, p38*β*, p38γ and p38*δ*. The first isoform p38*α* was identified in 1994 as a 38 kDa polypeptide that is activated in response to endotoxin treatment, cell stress or cytokines [23]. Three additional isoforms were described later: p38*β*, p38γ and p38*δ*. These kinases share highly similar protein sequences; p38*α* and p38*β* are 75% identical, whereas p38γ and p38*δ* are 62% and 61% identical to p38*α*, respectively. In turn, p38γ and p38*δ* are 70% identical to each other. The four p38MAPK isoforms are widely expressed, although p38*β*, p38γ and p38*δ* expression appear to be higher in specific tissues; for example, p38*β* is abundant in brain, p38γ in skeletal muscle, and p38*δ* in endocrine glands [23]. In general, all p38MAPKs are strongly activated by a wide variety of environmental and cellular stresses or by inflammatory cytokines and are poorly activated by serum or growth factors [23]. The canonical activation of p38 MAPKs occurs via dual phosphorylation of their Thr–Gly–Tyr motif, in the activation loop, by mitogen-activated protein kinase kinase (MKK) 3/6 (MKK3 and MKK6) [23]. Upon activation, the dually phosphorylated p38MAPK goes through characteristic global conformational changes that alters the alignment of the two kinase halves (N-terminal and C-terminal domains) of the folded protein and enhances access to the substrate, which together increases enzymatic activity. To date, most studies of the p38MAPK pathways focused on function of the p38α and p38*β* isoform, which is widely considered to negatively regulate malignant transformation; nonetheless, few reports address the p38γ and p38δ isoforms. Although p38γ and p38δ can phosphorylate typical p38 MAPK substrates such as the transcription factors ATF2, Elk-1 or SAP1, they cannot phosphorylate some substrates of p38*α* and p38*β* and have their unique substrates [23].

Recent studies indicate that p38γ may have some particular implications in breast cancer. For example, Meng et al. [30] showed that p38γ is overexpressed in highly metastatic human and mouse breast cancer cell lines and p38γ expression is preferentially associated with basal-like and metastatic phenotypes of breast tumor samples. Clinical evidence shows that elevated expression of p38γ is associated with lower overall survival of patients with breast cancer [24]. Using a computational mechanical model, Rosenthal et al further showed that p38γ can regulate the changes of cytoskeleton and cell shape of breast cancer cells and control cell motility. This evidence suggests an important role of p38γin the aggressiveness of breast cancer. However, it is unclear how p38γ is activated and how itregulates the aggressiveness of breast cancer cells. It is reported that p38γ can regulate RhoC expression by mediating RhoC protein stability through regulation of RhoC ubiquitination and lysosomal degradation [24]. We demonstrate that ethanol-induced activation of p38γinhibits the ubiquitination of RhoC, therefore increasing the stability of RhoC protein (Figure 10). More importantly, down-regulation of RhoC is sufficient to inhibit ethanol-induced migration and invasion in MCF7 cells. This suggests that p38γ and RhoC pathway plays an important role in ethanol-promoted aggressiveness of breast cancer.

It is currently unclear regarding the mechanisms of how ethanol specifically activates p38γ. One possibility is that the effect of ethanol is mediated through the production of reactive oxygen species (ROS). Mitogen-activated protein kinase kinase 6 (MKK6) is the major upstream kinase that activate p38γ and its activity is regulated by intracellular ROS concentration [31]. MKK6 activation in turn can enhance ROS production through NADPH oxidase [32]. Ethanol oxidation produces ROS. Although ethanol metabolism mainly occurs in the liver and is mediated by hepatic alcohol dehydrogenase IB (ADH1B), breast cancer cells are shown to express moderate levels of cytochrome P450 2E1, another enzyme to oxidize ethanol. We have previously demonstrated that ethanol increases intracellular ROS accumulation in breast cancer cells [15, 21]. Therefore, it is possible that ethanol activates ROS-p38γ-RhoC pathway which results in enhanced aggressiveness of breast cancer.

## MATERIALS AND METHODS

### Materials

Protein A/G beads were obtained from Santa Cruz Biotechnology (San Diego, CA). Polyclonal anti-phospho-p38 MAPK (Thr180/Tyr182) antibody was purchased from Life Technologies (Carlsbad, CA) and Cell Signaling Technology Inc. (Beverly, MA). Anti-p38α, p38β, p38γ MAPK and RhoC antibodies were purchased from Santa Cruz Biotechnology (San Diego, CA). Anti-GAPDH antibody was obtained from Research Diagnostics, Inc. (Concord, MA). MTT assay kit was purchased from Roche Molecular Biochemicals (Indianapolis, IN). p38γ shRNA, control shRNA, RhoC siRNA and control siRNA were purchased from Santa Cruz Biotechnology (San Diego, CA). Matrigel Matrix basement membrane and Matrigel Invasion Chambers were purchased from BD Biosciences (Bedford, MA). Transwell was obtained from Costar Corp. (Acton, MA). ALDEFLUOR kits were purchased from Stemcell Technologies (Vancouver, Canada). Antibiotic-Antimycotic (Anti-Anti) and cell culture mediums were obtained from Gibco (Life Technologies). All other chemicals were obtained from Sigma-Aldrich (St. Louis, MO).

### Cell culture and ethanol exposure

MCF7 and T47D were grown in DMEM medium containing 10% fetal bovine serum (FBS) and 1% Anti-Anti. BT20 cells were cultured in EMEM medium containing 10% FBS, 1% Anti-Anti, 3 mM glucose and 2 mM glutamine. SP-MCF7 (side population-MCF7) cells, a Hoechst dye excluding mammary cell subline, were a gift from Dr. Xiuwei Yang (University of Kentucky). Cells were maintained at 37°C with 5% CO_2_. A range of physiologically relevant concentrations of ethanol (0, 100, 200, or 400 mg/dl, i.e., 0, 22, 44 or 88 mM) was used in this study. Ethanol at 100 mg/dl (22 mM) was used in the most experiments. A method utilizing sealed containers was used to maintain ethanol concentrations in the culture medium. With this method, ethanol concentrations in the culture medium can be accurately maintained [33]. The containers were placed in a humidified environment and maintained at 37°C with 5% CO_2_.

### Generation of phospho-specific antibody against p38γ MAPK

Affinity-purified antibodies specifically against the dual-phosphorylation motif, Thr-Gly-Tyr [34] which is located in the activation loop [Thr(p) 180/Tyr(p) 182) on p38γ MAPK] were generated at 21st Century Biochemicals (Marlboro, MA). Rabbits were immunized with the phosphorylated p38γ peptide Acetyl-SEM[pT]G[pY]VVT-Ahx-C-amide and serum was affinity purified.

### Generation of cells stably expressing p38γ shRNA

To generate stable cell lines with silencing of p38γ, briefly, MCF7 cells were transfected with either scrambled short hairpin RNA (shRNA) as controls or shRNA for p38γ MAPK (Santa Cruz Biotechnology) using a Neon Transfection apparatus (Life Technologies). Cells expressing these shRNA were selected by the treatment of puromycin (4 μg/ml). Cell lysates were collected and the down-regulation of p38γ MAPK was confirmed by immunoblotting.

### Three-dimensional cell culture assay

The three-dimensional (3-D) cell culture system was established as previously described [35]. Briefly, 24-well plates were pre-coated with 150 μl Matrigel Matrix (BD Biosciences). Single cell suspension (10^3^ per well) were mixed with 200 μl ice-cold Matrigel and seeded in the pre-coated 24-well plates. After 30 min gelling, culture medium was added to the plates and the medium was changed every 2 to 3 days. The cell morphology was captured by a Zeiss Axiovert 40C photomicroscope.

### Immunoblotting and immunoprecipitation

Cells were lysed in modified RIPA buffer (150 mM NaCl, 50 mM Tris, 1% NP-40, 0.25% sodium deoxycholate) containing 1 mM sodium vanadate, 1 mM phenylmethanesulfonyl fluoride (PMSF), 5 μg/ml of aprotinin, and 2 μg/ml of leupeptin. The procedure for immunoblotting has been previously described (27). Briefly, protein samples were clarified by centrifugation at 14,000 rpm for 10 min at 4°C and resolved by sodium dodecyl sulfate-polyacrylamide gel electrophoresis (SDS-PAGE). The separated proteins were transferred to nitrocellulose membranes. The membranes were probed with the indicated primary antibodies, followed by the appropriate horseradish peroxidase-conjugated secondary antibodies, and developed by enhanced chemiluminescence.

For immunoprecipitation, equal amount of proteins (about 500–800 μg) were incubated with anti-p38γ, p38α, phosphorylated-p38 (p-p38), phosphorylated-p38γ (p-p38γ) or ubiquitin antibodies at 4°C overnight. The protein/antibody complex was treated with Protein A/G beads conjugated to agarose at 4°C for 4 hours, and then centrifuged at 5,000 g for 5 min. Samples were washed 5 times with RIPA buffer, 1 time with cold-TBS, and then boiled in sample buffer (187.5 mM Tri-HCl, pH 6.8, 6% SDS, 30% glycerol, 150 mM DTT and 0.03% bromophenol blue). Proteins were resolved in SDS PAGE and analyzed by immunoblotting.

### MTT assay

The MTT assay was performed to determine the number of viable cells as previously described [21]. Briefly, equal amount of cells were plated into 96-well plates and cultured for 24 hours. After that, 10 μl of MTT reagent was added to each well and the plates were incubated at 37°C for 4 hours. The cultures were solubilized and spectrophotometric absorbance was measured at 595 nm using a microtiter platereader (Beckman coulter).

### Cell invasion and migration

Cell invasion was assayed using Matrigel Invasion Chambers (BD Biosciences). Cell migration was analyzed using a Transwell Migration System (Costar). Briefly, equal amount of cells were placed on the upper compartment of invasion chambers or Transwell chambers (8.0 μm pore size) which contained serum-free medium. Culture medium containing 10% FBS was added to the lower compartment of invasion/migration chambers to serve as chemoattractants. The chambers were cultured at 37°C in 5% CO_2_ in the presence or absence of ethanol (100 mg/dl) for 12 hours. The invaded/migrated cells were fixed in 4% paraformaldehyde and stained with 0.5% crystal violet in 2% ethanol. Membranes were washed and the dye was eluted with 10% acetic acid. Absorbance was measured at 595 nm using a microtiter plate reader (Beckman coulter).

### Anchorage-independent colony formation

Anchorage-independent colony formation was assayed as previously described [36]. Briefly, 5,000 cells were mixed with 0.3% agar in culture medium. The mixture was plated on the top of 1% agar in six-well plates with an addition of 1 ml of medium. The medium was changed every 3 to 4 days. The cultures were incubated for a month at 37°C in 5% CO_2_, and stained with 0.5 ml of 0.005% Crystal Violet. Colonies (diameter ≥ 0.1 mm) were counted using a dissecting microscope, and images were captured using a Zeiss Axiovert 40C photomicroscope.

### Assaying Stem-like cell population assay

The stem-like tumor cells were identified by measuring aldehyde dehydrogenase (ALDH) activity (ALDEFLUOR assay). The ALDEFLUOR assay was performed according to the manufacturer's protocol (Stemcell Technologies). ALDH enzymatic activity was determined by flow cytometry as previously described [37, 38]. Briefly, after chronic exposure to ethanol (0, 100 or 200 mg/dl), cells were collected and 10^6^ cells were incubated in ALDEFLUOR assay buffer containing ALDH substrate (1 μmol/l per 1 × 10^6^ cells) for 40 minutes at 37°C. Some cells were treated under identical conditions with a specific ALDH inhibitor (50 mmol/l, diethylaminobenzaldehyde) as a negative control. Cells were sorted using a flow cytometer (FACSCalibur, Becton Dickinson) and analyzed using the WINMDI software. The results were presented relative to the controls.

### siRNA and cell transfection

Transient transfection of RhoC siRNA (RhoC siRNA) or scrambled siRNA (Con siRNA) (San Cruz Biotech) was performed using a Neon Transfection System (Invitrogen Corporation, Carlsbad, CA) according to the manufacturer's protocol. Briefly, MCF7 cells were exposed to ethanol (0 or 100 mg/dl) for 10 days, and then electroporated with Con siRNA or RhoC siRNA using a Neon transfection apparatus. After electroporation, cells were incubated for 48 hours, and then assayed for cell migration/invasion in the presence or absence of ethanol (100 mg/dl).

### Xenograft tumor model

Ten week-old athymic nude (NU/NU) mice (Harlan Laboratories) were used in this experiment. All procedures were performed in accordance with the guidelines set by the National Institutes of Health (NIH) Guide for the Care and Use of Laboratory Animals and were approved by the Institutional Animal Care and Use Committee. To test the tumorigenicity, MCF7 cells (10^3^ - 10^6^ cells/100 μl PBS) that were exposed to ethanol (0 or 100 mg/dl) were subcutaneously inoculated into both sides of the lower flank of nude mice. For each group, there were six mice. One month after the inoculation, tumors in each mouse were determined. To determine the effect of p38γ MAPK knock-down on the growth of tumors, MCF7 stably expressing control shRNA or p38γ shRNA were exposed to ethanol (0 or 100 mg/dl) for a month. These cells (5 × 10^6^ cells/100 μl PBS) were inoculated in to nude mice. Tumor size was measured weekly using a caliper. Tumor volumes were calculated using the formula: volume (mm^3^) = (length x width^2^)/2. Mice were euthanized approximately one month after injection. Metastases in lung were analyzed as previously described [18].

### Immunohistochemical staining

Immunohistochemical (IHC) staining was performed as described [18]. Briefly, tumor tissues and lungs were removed and fixed with 4% paraformaldehyde and then transferred to 30% sucrose. Tissues were sectioned at 10 μm thickness using a Cryostat Microtome (Thermo Scientific). Tumor tissues were stained with phosphorylated p38γ using Diaminobenzidine (DAB) kit. Briefly, tissues were incubated with 0.3% H_2_O_2_ in methanol for 30 min at room temperature and then treated with 0.1% TritonX-100 for 10 min. The sections were washed twice with PBS and then blocked with 1% BSA and 0.05% TritonX-100 for 1 hour. The sections were incubated with phospho-p38γ antibody (1:300) overnight at 4°C. Negative controls were performed by omitting the primary antibody. After being rinsed in PBS, sections were incubated with a biotinylated goat anti-rabbit IgG (Vector Laboratories, Burlingame, CA) for an hour at room temperature. The sections were washed three times with PBS and incubated with in an avidin-biotin-peroxidase complex (1:100) for an hour and developed in 0.05% 3,3′- Diaminobenzidine (Invitrogen) containing 0.003% H_2_O_2_ in PBS. To examine the lung metastasis, the sections of lung tissues were stained with eosin and images were recorded using an Olympus BX51 microscope.

### Statistics

Differences among treatment groups were analyzed using analysis of variance (ANOVA). Differences in which *p* was less than 0.05 were considered statistically significant. In cases where significant differences were detected, specific *post-hoc* comparisons between treatment groups were examined with Student-Newman-Keuls tests. The prevalence of lung metastasis between control and alcohol-treated groups was determined by the Fisher exact test.
